# Axillary management for early invasive breast cancer patients: Who will truly benefit?

**DOI:** 10.3389/fonc.2022.989975

**Published:** 2022-08-15

**Authors:** Yanbiao Liu, Yan Fan, Zining Jin, Mengyao Cui, Xinmiao Yu, Feng Jin, Xu Wang

**Affiliations:** ^1^ Department of Breast Surgery, The 1st Affiliated Hospital, China Medical University, Shenyang, China; ^2^ Department of Pediatrics, The First Hospital of China Medical University, Shenyang, China; ^3^ Department of Cell Biology, Key Laboratory of Cell Biology, Ministry of Public Health, Key Laboratory of Medical Cell Biology, Ministry of Education, China Medical University, Shenyang, China; ^4^ Research Unit of General Surgery, Department of Breast Surgery and Surgical Oncology, The First Hospital of China Medical University, Shenyang, China

**Keywords:** surgery, invasive breast cancer, sentinel lymph node biopsy, completion axillary lymph node dissection, metastasis

## Abstract

**Background:**

The implementation of sentinel lymph node biopsy (SLNB) and further completion axillary lymph node dissection (cALND) after positive sentinel lymph nodes (SLNs) on early invasive breast cancer patients should be cautiously tailored. Identifying predictors for SLN and non-sentinel lymph node (nSLN) metastases can help surgeons make better surgical decisions.

**Methods:**

A retrospective case-control study was designed and a total of 560 eligible patients were enrolled consecutively. They were all diagnosed in our center and received appropriate medical care. According to the metastasis of SLN and nSLN, they were divided into metastatic and non-metastatic groups on two successive occasions to investigate the relationship between clinical factors, pathological factors, hematological factors and lymph node metastasis.

**Results:**

In total, 101 (18.04%) patients developed SLN metastases, including 98 patients with macro-metastases and 3 patients with micro-metastases. Out of 97 patients receiving further cALND, 20 patients (20.62%) developed nSLN metastases. Multivariate analysis revealed that “high expression of Ki-67” and “lymphatic invasion” predicted a higher risk of SLN metastasis; and “increased number of positive SLNs” and “increased systemic inflammation index (SII)” predicted a higher risk of nSLN metastasis.

**Conclusion:**

Surgery for early invasive breast cancer patients should be more customized and precise. Appropriate axillary management is necessary for patients with the associated predictors.

## Introduction

Axillary management of early invasive breast cancer patients contributes to a favorable prognosis by attaining local control and obtaining information on postoperative systemic therapy decisions (1). SLNB has been authorized a safe and reliable alternative to one-stage ALND for assessing axillary lymph node status in clinically node-negative breast cancer patients (2). For patients with negative SLNs, SLNB alone can attain satisfactory local control and a non-inferior prognosis compared with cALND while avoiding serious side-effects, thus improving the patient’s quality of life (3). On the other hand, in patients with positive SLNs, SLNB provides surgeons with information to decide the extent of further surgical resection (4). And for such patients, cALND has long been considered the gold standard (5).

However, this notion has now been challenged. Do we really need cALND in all patients with positive SLNs? Existing studies revealed that less than one-third of SLN-positive patients had nSLN metastases, implying that for most patients, cALND was not able to contribute to a better local control at a cost of heavier financial pressure and more serious side-effects (6). Meanwhile, as the understanding of tumor biology deepens, the postoperative systemic treatment decision is based more on molecular typing and genetic patterns, resulting in a decrease in the impact of information obtained from cALND (7). According to the ACOSOG Z0011 trial and the IBCSG 23-01 trial, cALND could be conditionally omitted in patients with limited disease in SLNs, and the American Society of Clinical Oncology (ASCO) Expert Panel have made pertinent recommendations on this top (8–11). However, this strategy has been criticized due to the high rates of micro-metastasis and rigorous enrollment requirements of the cornerstone studies, which were believed to reduce the persuasiveness (12).

Given the trend towards minimizing intrusive surgical procedures, SLNB also loses much of its importance. Since SLN-negative patients have a satisfactory prognosis; further cALND makes a limited contribution to local control; and the impact of lymph node status on systemic treatment decisions is declining, do we really need to perform SLNB in all invasive breast cancer patients? (9, 13, 14)

Axillary management of breast cancer patients should be more customized and precise. If we can screen out patients with only SLN metastasis, unnecessary surgeries could be avoided. In this study, we retrospectively reviewed 560 patients’ electronic medical records. Clinical, pathological and hematological constants were analyzed to explore potential predictors for SLN and nSLN metastasis.

## Methods

### Study design

We designed a retrospective case-control study to investigate which factors were independently associated with SLN metastasis and further nSLN metastasis in SLN-positive patients. All enrolled patients were divided into SLN metastasis group and SLN non-metastasis group for the first analysis. Then, within the SLN metastasis group, eligible patients were further divided into nSLN metastasis group and nSLN non-metastasis group for the second analysis. All enrolled patients received standard surgical treatment as recommended by the national comprehensive cancer network (NCCN) Clinical Practice Guidelines (15).

### Inclusion and exclusion criteria

Inclusion criteria: Female patients diagnosed with invasive breast cancer and underwent SLNB from December 2019 to December 2021 at the Department of Breast Surgery, The First Affiliated Hospital of China Medical University. Exclusion criteria: (1) Male patients (2) Patients with pathologically diagnosed pure ductal carcinoma in situ, lobular carcinoma in situ, encapsulated papillary carcinoma or Paget’s disease. (3) Patients who have received neoadjuvant chemotherapy (NAC) or are proposed to receive neoadjuvant chemotherapy. (4) Patients with inflammatory or blood disorders. (5) Patients who are taking drugs able to affect the results of hematology tests, including antibiotics, anti-inflammatory drugs and anticoagulants.

### Operating methods

The single tracer method was adopted for all patients, with nanocarbon or methylene blue as the tracer. Methylene blue was injected intracutaneously or subcutaneously around the ipsilateral areola 5-15 minutes prior to surgery. Nanocarbon was injected in the same way, 6-8 hours before surgery (16, 17). SLN refers to one or a few lymph nodes to which breast cancer cells metastasize at first (5). Frozen sections of the SLNs were used for intraoperative pathology examination, and remaining tissues from the section were paraffin-embedded for further pathological examination. Macro-metastases were defined as tumor deposits with a maximum diameter >2 mm. Micro-metastases were defined as tumor deposits with a maximum diameter >0.2 mm and ≤2 mm or over 200 tumor cells being seen in one frozen section. Both macro- and micro-metastases of sentinel lymph nodes were considered positive. The presence of isolated tumor cells or absence of tumor cells in frozen sections was considered negative (18).

### Diagnosis of lesions

The pathological types of tumors were classified as invasive ductal carcinoma, invasive lobular carcinoma and others, including septate carcinoma, mucinous carcinoma, invasive papillary carcinoma, invasive micropapillary carcinoma, septate carcinoma and carcinoma with neuroendocrine differentiation. Immunohistochemical markers estrogen receptors (ER), progesterone receptors (PR), human epidermal growth factor receptor 2 (HER2) and Ki-67 were detected and interpreted as specified (19, 20). For ER and PR, 10% was regarded as the threshold to distinguish between high expression and low/no expression groups. For HER2, 3+ was considered the label of high expression group, and 0+, 1+ and 2+ were considered the labels of low/no expression group. For Ki-67, 30% was regarded as the threshold to distinguish between high expression and low/no expression groups (21, 22). The unifocal and multifocal nature of lesions was evaluated preoperatively and confirmed intraoperatively. The location of lesions was divided into upper outside quadrant and other quadrants, including upper inside quadrant, lower inside quadrant, lower outside quadrant and central area, due to the character of upper outside quadrant as the most frequent location of breast cancers, also seeming to imply that tumors in this area are more likely to metastasize to the axillary region (23, 24).

### Collection of blood samples

All blood samples were obtained within one week prior to surgery. If two sets of hematological data were available, the one closer to the date of surgery would be used for analysis to ensure the representativeness. We recorded platelet count, neutrophil count, lymphocyte count and calculated three parameters including platelet lymphocyte ratio (PLR), neutrophil lymphocyte ratio (NLR) and systemic inflammation index (SII) on this basis. PLR was calculated as platelet/lymphocyte. NLR was calculated as neutrophil/lymphocyte. And SII was calculated as platelet × neutrophil/lymphocyte.

### Statistical analysis

Age was the only continuous variable conforming to a normal distribution tested by the Shapiro-Wilk method and was presented as the form of mean ± standard deviation. Other continuous variables were presented as median joint quartiles. The intergroup difference in average of age was comprised by Student’s t-test. Intergroup differences in non-normally distributed continuous variables and hierarchical variables were compared by Mann Whitney-U test. Intergroup differences in counting variables were comprised by chi-square test. Independent predictors for metastasis were determined by the logistic regression model. Variables with *p*<0.1 in univariate analysis were enrolled in multivariate analysis. All analyses were two-tailed with 0.05 as the statistical threshold. The SPSS version 26.0 was used for all statistical analysis.

## Results

### Participants and lymph node metastasis

**Figure 1:**
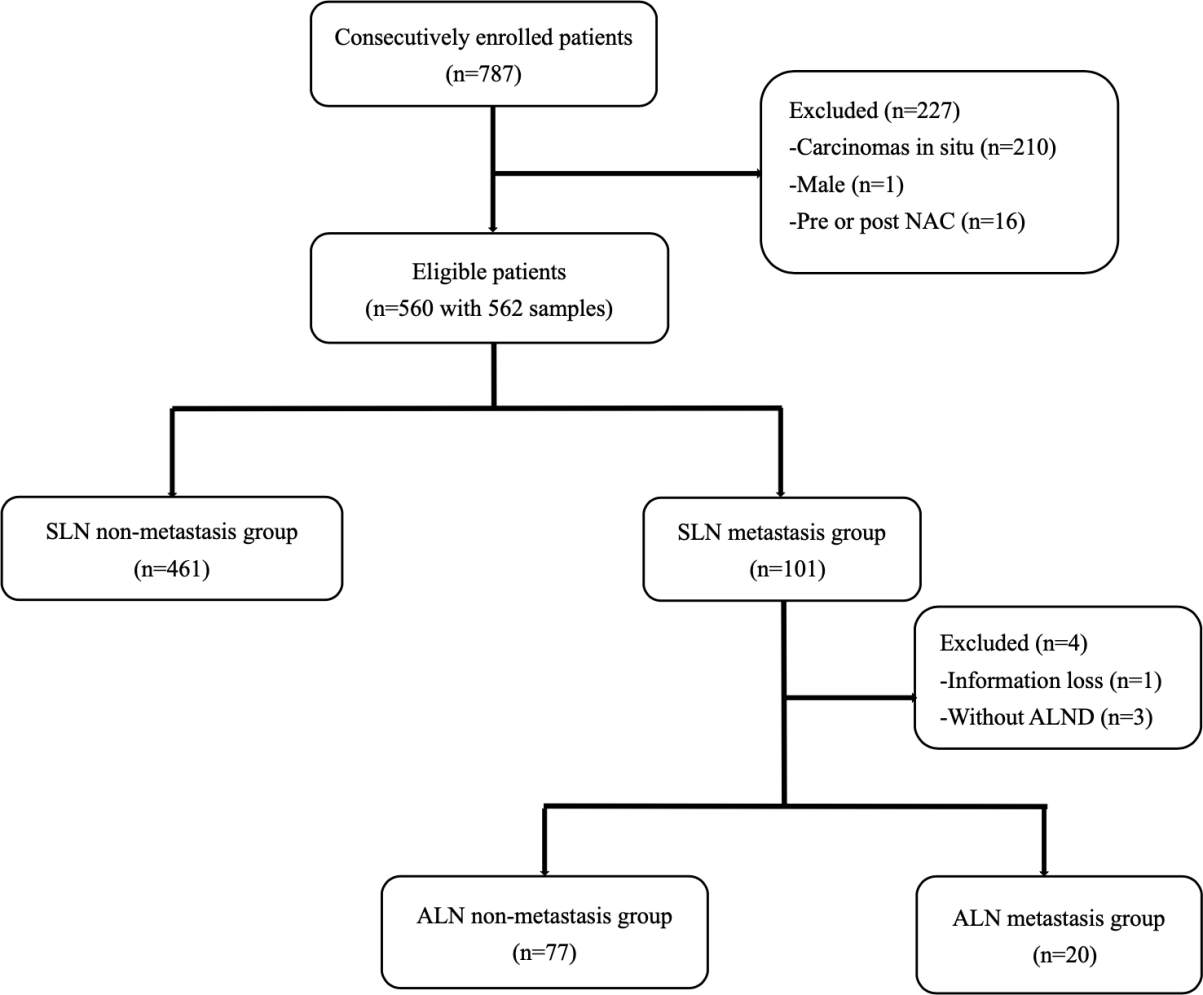
Study design and population.

A total of 787 breast cancer patients received SLNB in our center from December 2019 to December 2021. After screening, 562 samples from 560 patients who met the inclusion criteria were consecutively included (**Figure 1**). The mean age of all eligible patients was 51.79±10.54. Two patients received bilateral SLNB, and they were both divided into the non-metastasis group. All patients were staged cT1-2N0M0 according to the Eighth Edition of AJCC Cancer Staging Manual (25).

In total, 101 patients developed SLN metastases, accounting for 18.04%. Macro-metastases were found in 98 patients, accounting for 97.03%. Micro-metastases were found in only three patients, accounting for 2.97%, and none of them had nSLN metastasis. A total of 97 patients in the metastasis group received cALND, and 20 of them had nSLN metastases, accounting for 20.62%. Nine patients had ≥3 positive SLNs, of whom 4 patients had nSLN metastases, accounting for 44.44%. While in the remaining 88 patients with 1-2 positive SLNs, nSLN metastases were found in 16 patients, accounting for 18.18%.

### Baseline characteristics

The mean age of patients in SLN metastasis and non-metastasis groups was comparable (50.88 ± 9.39 vs 51.99 ± 10.77, *p*=0.34), accompanied by a similar distribution of menstrual status (*p*=0.90). The difference in body mass index (BMI) was not statistically significant (24.20 vs 24.00, *p*=0.28). A minority of patients in both groups have family history of breast cancer (8.16% in metastasis group and 7.26% in non-metastasis group, *p*=0.76).

Patients in the metastasis group had statistically significant larger lesions than those in non-metastasis group (2.30 cm vs 2.00 cm, *p*=0.01). In both groups, mono-focal lesions were in the majority and the tiny intergroup differences were not statistically significant (86.87% vs 90.79%, *p*=0.24). For other properties of the lesion, including anatomic subdivisions (*p*=0.67), locations (*p*=0.91) and palpability (*p*=0.41), intergroup differences were minor and without statistical significance.

In the metastatic group, 69(68.32%) patients received mastectomy and 32(31.68%) patients received breast conserving surgery (BCS). Methylene blue was used as the tracer in 95 patients (94.06%) and nanocarbon in 6 patients (5.94%). An average of 4 SLNs were resected intraoperatively. In the non-metastatic group, 278(60.30%) patients received mastectomy and 183(39.70%) patients received BCS. Methylene blue was used as the tracer in 441 patients (95.67%) and nanocarbon in 6 patients (4.33%). An average of 3 SLNs were resected intraoperatively. None of the intergroup differences were statistically significant (*p*=0.13 for breast surgery, *p*=0.49 for tracer and *p*=0.18 for SLN number).

When it comes to pathological type and histological grade, “Invasive ductal carcinoma” and “Grade II” were in the majority in both groups (90.1% of “Invasive ductal carcinoma” and 89.36% of “Grade II” in the metastatic group; 84.75% of “Invasive ductal carcinoma” and 72.68% of “Grade II” in the non-metastatic group) and no statistically significant intergroup differences were noticed (*p*=0.22 for pathological type and *p*=0.28 for histological grade). There were more patients with high expression of ER and PR in the metastatic group compared to those in the non-metastatic group (91.00% vs 79.87% for ER, *p*<0.01 and 82.00% vs 70.35% for PR, *p*=0.02). As for the expression of Ki-67, although low-expressing patients occupied the majority in both groups (58.00% in the metastasis group and 68.36% in the non-metastasis group), the proportion of high-expressing patients in the metastatic group was significantly higher than those in the non-metastatic group (42.00% vs 31.64%, *p*=0.04). The expression of HER2 was comparable (*p*=0.04). In addition, a higher percentage of “Lymphatic invasion” and “Nerve invasion” was noticed in the metastasis group (13.51% vs 2.56% for Lymphatic invasion, *p*<0.01 and 8.11% vs 2.28% for Nerve invasion, *p*=0.02). No statistically significant intergroup differences in hematological constants were noticed (*p*=0.74 for PLR, *p*=0.69 for NLR and *p*=0.68 for SII) (**Table 1**).

**Table 1:** Characteristics between SLN metastasis and SLN non-metastasis groups.

Characteristics	SLN metastasis group (n = 101)	SLN non-metastasis group (n = 461)	*p*
Age (n = 560)	50.88 ± 9.39	51.99 ±10.77	0.34
BMI (n = 560)	24.20 (22.18, 26.73)	24.00 (21.97, 25.95)	0.28
Menstruation (n = 546)			
Pre-menopause	51 (51.52%)	227 (50.78%)	
Menopause	48 (48.48%)	220 (49.22%)	0.90
Family history of BC (n = 539)			
Without	90 (91.84%)	409 (92.74%)	
With	8 (8.16%)	32 (7.26%)	0.76
Lesion size (cm) (n = 544) Number of lesions (n = 544) Mono-focal Multi-focal Anatomic subdivisions (n = 562)	2.30 (1.70,2.86) 86 (86.87%) 13 (13.13%)	2.00 (1.50,2.70) 404 (90.79%) 41 (9.21%)	0.01 0.24
Left	50 (49.50%)	239 (51.84%)	
Right	51 (50.50%)	222 (48.16%)	0.67
Locations (n = 547)			
Superior-lateral quadrant Others	46 (46.94%) 52 (53.06%)	208 (46.33%) 241 (53.67%)	0.91
Palpability (n = 544)			
Palpable	93 (93.94%)	407 (91.46%)	
Impalpable	6 (6.06%)	38 (8.54%)	0.41
Breast surgery (n = 562)			
Mastectomy	69 (68.32%)	278 (60.30%)	
BCS	32 (31.68%)	183 (39.70%)	0.13
Tracer (n = 562) Methylene blue Nanocarbon	95 (94.06%) 6 (5.94%)	441 (95.67%) 20 (4.33%)	0.49
Number of SLN (n = 561)	4.00 (3.00,5.00)	3.00 (2.00,5.00)	0.18
Pathological type (n = 560)			
Invasive ductal carcinoma	91 (90.10%)	389 (84.75%)	
Invasive locular carcinoma	6 (5.94%)	28 (6.10%)	
Others	4 (3.96%)	42 (9.15%)	0.22
Histological grade (n = 503)			
Grade I	1 (1.07%)	29 (7.08%)	
Grade II	84 (89.36%)	298 (72.68%)	
Grade III	9 (9.57%)	83 (20.24%)	0.28
ER (n = 552)			
High expression	91 (91.00%)	361 (79.87%)	
Low or no expression	9 (9.00%)	91 (20.13%)	<0.01
PR (n = 552)			
High expression	82 (82.00%)	318 (70.35%)	
Low or no expression	18 (18.00%)	134 (29.65%)	0.02
HER2 (n = 552)			
High expression	10 (10.00%)	49 (10.62%)	
Low or no expression	90 (90.00%)	404 (89.38%)	0.86
Ki-67 (n = 551)			
High expression	42 (42.00%)	142 (31.64%)	
Low expression	58 (58.00%)	309 (68.36%)	0.04
Lymphatic invasion (n = 425)			
With	10 (13.51%)	9 (2.56%)	
Without	64 (86.49%)	342 (97.44%)	<0.01
PLR (n = 501)	131.87 (101.32, 168.70)	133.14 (105.91, 166.84)	0.74
NLR (n = 501)	1.74 (1.34, 2.29)	1.70 (1.29, 2.29)	0.69
SII (n = 501)	430.73 (289.29, 581.25)	418.53 (292.65, 582.83)	0.98

### Independent predictors for SLN metastasis

**Figure 2:**
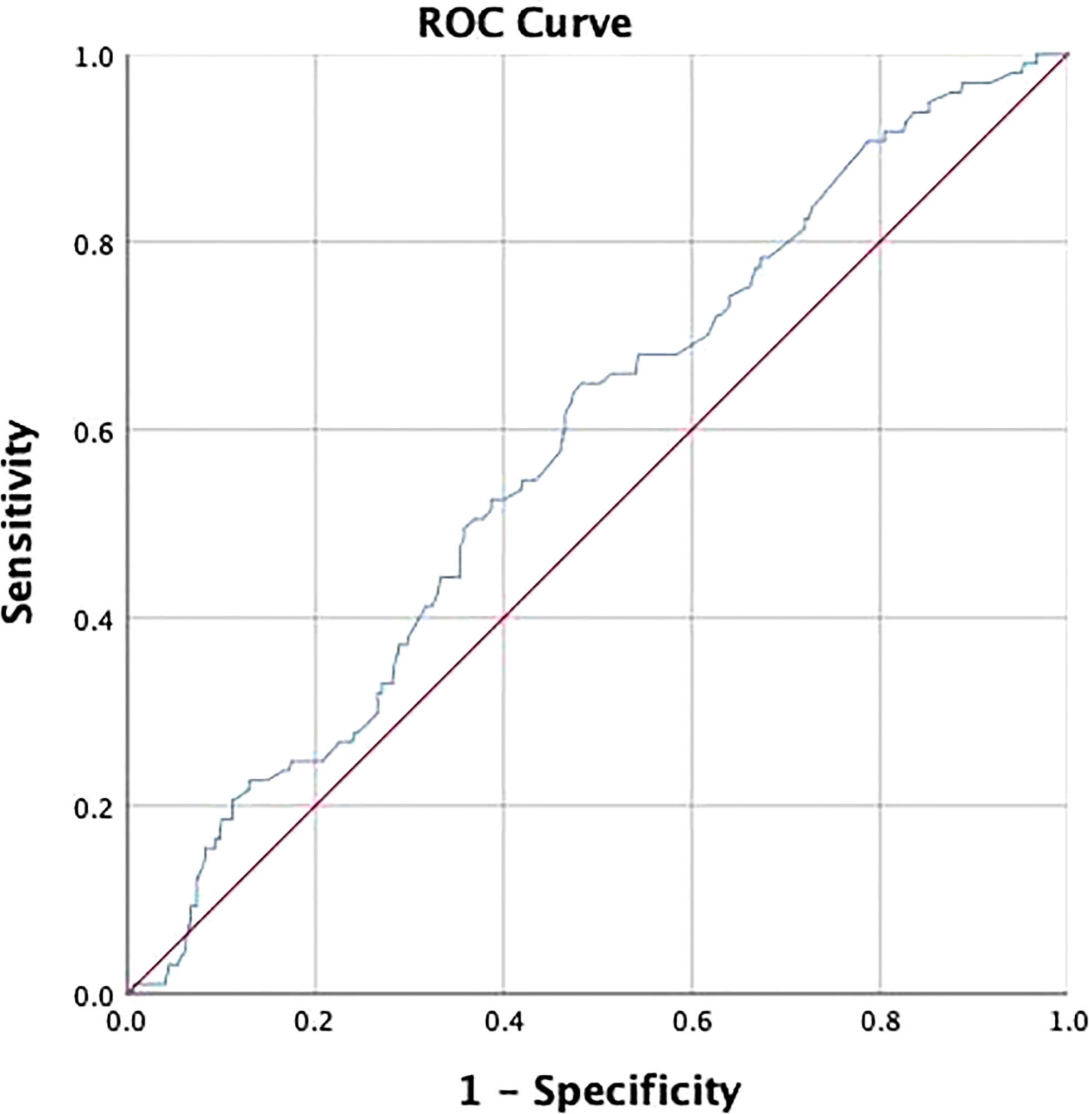
Receiver operating characteristic (ROC) curve was used to determine the cut-off value of lesion size and assess its discriminative power. The cut-off value was 3.25 and the area under the curve (AUC) was 0.583 (P = 0.01).

The receiver operating characteristic (ROC) analysis was adopted to determine the cut-off value of lesion size and assess its discriminative power. The cut-off value was 3.25 and the area under the curve (AUC) was 0.583 (*p*=0.01) (**Figure 2**). The expression of Ki-67 (odds ratio, 2.52; confidence interval, 0.67–7.58; *p*<0.01) and lymphatic invasion (odds ratio, 4.12; confidence interval, 1.35–12.97; *p*=0.01) were established as independent predictors for SLN metastasis after univariate and multivariate logistic regression analysis (**Table 2**).

**Table 2:** Logistic regression for lesion size, lymphatic invasion, nerve invasion, ER, PR and Ki-67.

	Univariate analysis		Multivariate analysis	
	OR (95%CI)	p	OR (95%CI)	*p*
Lesion size	1.21 (1.00,1.48)	0.06	1.14 (0.89,1.45)	0.26
ER (>10% vs ≤10%) PR (>10% vs ≤10%) Ki-67 (>30% vs ≤30%)	2.55 (1.24,5.25) 1.92 (1.11,3.32) 1.58 (1.01,2.46)	0.01 0.02 0.05	2.24 (0.67,7.52) 2.22 (0.82,5.98) 2.50 (1.43,4.36)	0.16 0.14 <0.01
Lymphatic invasion (with vs without)	5.94 (2.32,15.19)	<0.01	4.23 (1.37,13.07)	<0.01

### Subgroup comparisons and the independent predictors for nSLN metastasis

A total of 4 patients were excluded from the second analysis in the SLN metastasis group, including one with missing information and three without further cALND. Based on whether nSLN metastases were detected, 20 of all eligible patients were divided into the metastatic group and the remaining 77 patients were divided into the non-metastatic group.

Patients in the nSLN metastasis group had more SLN metastases than those in the non-metastasis group (2.00 vs1.00, *p*<0.01). Besides, the analysis of hematological parameters revealed statistically significant intergroup difference in the expression of SII (599.83 vs 405.91, *p*=0.02) (**Table 3**). No statistically significant intergroup differences were noticed in other patient characteristics, lesion characteristics, surgical approaches and nature of the pathology. However, it was worth mentioning that patients in the metastatic group were younger than those in the non-metastatic group (49.15 vs 51.36, *p*=0.10). Patients in the metastatic group had larger lesion size compared with those in the non-metastatic group (2.39 cm vs 2.23 cm, *p*=0.68).

**Table 3:** Characteristics between nSLN metastasis and nSLN non-metastasis groups.

Characteristics	nSLN metastasis group (n = 20)	nSLN non-metastasis group (n = 77)	*p*
Age (n = 97)	49.15 ± 9.09	51.36 ± 9.68	0.36
BMI (n=97)	24.93 (22.73, 27.77)	24.03 (22.05, 26.19)	0.30
Menstruation (n = 95)			
Pre-menopause	12 (60.00%)	36 (48.00%)	
Menopause	8 (40.00%)	39 (52.00%)	0.34
Family history of BC (n = 95)			
Without	16 (84.21%)	71 (93.42%)	
With	3 (15.79%)	5 (6.58%)	0.41
Lesion size (cm) (n = 93) Number of lesions (n = 95) Monofocal Multifocal Anatomic subdivisions (n = 97)	2.39 (1.90,3.15) 20 (100.00%) 0 (0.00%)	2.23 (1.69,2.96) 62 (82.67%) 13 (17.33%)	0.68 0.10
Left	11 (55.00%)	37 (48.05%)	
Right	9 (45.00%)	40 (51.95%)	0.58
Locations (n=94)			
Superior-lateral quadrant Others	11 (55.00%) 9 (45.00%)	35 (47.30%) 39 (52.70%)	0.54
Palpability (n = 95)			
Palpable	19 (95.00%)	70 (93.33%)	
Impalpable	1 (5.00%)	5 (6.67%)	1.00
Breast surgery (n = 97)			
Mastectomy	13 (65.00%)	55 (71.43%)	
BCS	7 (35.00%)	22 (28.57%)	0.58
Tracer (n = 97) Methylene blue Nanocarbon	19 (95.00%) 1 (5.00%)	73 (94.81%) 4 (5.19%)	1.00
Number of metastatic SLNs (n = 97)	2.00 (1.00,2.00)	1.00 (1.00,2.00)	<0.01
Pathological type (n = 97)			
Invasive ductal carcinoma	17 (85.00%)	70 (90.91%)	
Invasive locular carcinoma	2 (10.00%)	4 (5.19%)	
Others	1 (5.00%)	3 (3.90%)	0.73
Histological grade (n = 90)			
Grade I	0 (0.00%)	1 (1.38%)	
Grade II	15 (83.33%)	67 (93.06%)	
Grade III	3 (16.67%)	4 (5.56%)	0.10
ER (n=96)			
High expression	18 (90.0%)	69 (90.79%)	
Low or no expression	2 (10.00%)	7 (9.21%)	1.00
PR (n = 96)			
High expression	17 (85.00%)	61 (80.26%)	
Low or no expression	3 (15.00%)	15 (19.74%)	0.87
HER2 (n = 96)			
High expression	1 (5.00%)	8 (10.53%)	
Low or no expression	19 (95.00%)	68 (89.47%)	0.75
Ki-67 (n=96)			
High expression	11 (55.00%)	28 (36.84%)	
Low expression	9 (45.00%)	48 (63.16%)	0.14
Lymphatic invasion (n = 70)			
With	2 (11.76%)	7 (13.21%)	
Without	15 (88.24%)	46 (86.79%)	1.00
PLR (n = 92)	133.58 (112.69, 176.10)	132.84 (100.86, 169.23)	0.34
NLR (n = 92)	1.83 (1.62, 2.54)	1.69 (1.33, 2.28)	0.14
SII (n = 92)	599.83 (359.39, 718.51)	405.91 (275.25, 525.76)	0.02

After univariate and multivariate logistic analysis, both ‘number of metastatic SLN’ (odds ratio, 2.59; confidence interval, 1.34–4.99; P<0.01) and ‘SII’ (odds ratio, 1.003; confidence interval, 1.001–1.005; P=0.01) were determined independent predictors for nSLN metastasis (**Table 4**).

**Table 4:** Logistic regression for Number of metastatic SLN and SII. .

	Univariate analysis		Multivariate analysis	
	OR (95%CI)	p	OR (95%CI)	*p*
Number of metastatic SLNs	2.16 (1.20,3.88)	0.01	2.59 (1.34,4.99)	<0.01
SII	1.002 (1.000,1.004)	0.03	1.003 (1.001,1.005)	0.01

## Discussion

Axillary management has been considered indispensable for breast cancer patients although the scope of surgery is still up for debate (1). Patients tend to have smaller tumors and lower axillary burdens as a result of early diagnosis and advances in imaging techniques (26). With this comes the constant de-escalating of surgical procedure, and SLNB is a significant milestone.

SLNB entails preoperative injection of a tracer and intraoperative excision of SLNs for pathological examination; if the result is negative, further cALND is eliminated. However, in case of a positive result, the need for further cALND is debated.

The role of cALND was challenged because of the low rate of nSLN metastasis in SLN-positive patients and the serious accompanied side-effects. In the Z0011, IBCSG 23-01 and AMAROS trials, the rate of nSLN metastasis was 27.3%, 13% and 33% respectively (6, 10, 27). On the other hand, the long-term follow-up results of NSABP B-32 trial revealed that incidence of upper limb lymphedema in patients receiving cALND was 4 times higher than those receiving SLNB alone (28). In our study, a total of 97 SLN-positive patients received cALND, and only 20 of them were with nSLN metastases, accounting for 20.6%.

Furthermore, the safety of eliminating cALND seemed to be proven. The Z0011 trial reveled that for cT1-2N0M0 breast cancer patients with 1-2 positive SLNs, cALND contributed neither to a better local control nor to a longer disease-free survival (DFS) or overall survival (OS) if patients received BCS, systemic therapy, or radiotherapy. The IBCSG 23-01 trial reached similar results after 10-years follow-up. Besides, the AMAROS trial confirmed the safety of radiotherapy in place of cALND.

Noteworthily, in the 3 clinical trials mentioned, the percentage of patients with SLN micro-metastases was 41.2%, 28.8% and 98% respectively, which was also the reason why they were criticized. It was well known that patients with micro-metastasis had a better outcome than those with macro-metastasis (29). However, there are still a large number of patients with SLN macro-metastases in the clinic. In our study, there were 98 patients with macro-metastases out of 101 SLN-positive patients, accounting for 97%. It would be irresponsible to omit cALND in such patients who are ineligible for present clinical trials.

Concerns regarding the need for cALND have led to questions about the role of SLNB. Is it really necessary to carry out SLNB in all clinically node-negative patients? First, the rate of SLN metastasis is extremely low, ranging from 15% to 35% (30–32). Second, although being reduced, side-effects of SLNB were still present. Hanne Verbelen et al. followed 126 SLN-negative patients for 7 years and discovered that one-quarter of them suffered from arm and shoulder complaints (33). Gebruers et al. reviewed 28 articles and found that lymphedema was still a problem with 0%-63% incidence for SLN-negative patients (34). In our study, only 101 patients discovered SLN metastases, accounting for 18.04%.

These considerations suggested that the implementation of SLNB and further cALND should be tailored more cautiously. In our study, 4 independent predictors for SLN and nSLN metastases were identified.

Ki-67, a proliferation marker, has been commonly used as a prognosis and treatment selection signal despite substantial inter-laboratory variability (35). Breast cancer patients with high Ki-67 expression have been proven to have a higher incidence of distant metastasis and recurrence, as well as a worse overall survival (21). Surprisingly, Ki-67 isn’t included in any of the common nomograms for predicting non-sentinel metastasis after a positive SLN (36). Similarly, our study suggested that high expression of Ki-67 was an independent predictor for SLN metastasis. However, it did not show statistical relevance for nSLN metastases after a positive SLN, coinciding with existing studies.

Lymphatic invasion is one of the most essential steps in cancer cell metastasis, and it has been linked to a poorer DFS and OS for breast cancer patients (37). SLN-positive patients with lymphatic invasion have a higher risk of further metastasis, according to a study by Kimberly J. Van Zee et al. (38) Similarly, patients with lymphatic invasion had a higher chance of SLN metastases in our study.

The number of positive SLNs has been identified as a determinant in surgical decision-making and prognosis evaluation. The cALND can be skipped in breast cancer patients with 1-2 positive SLNs, according to ASCO guidelines (11). In our study, patients with ≥3 positive SLNs had a nSLN metastasis rate of 40%, which was considerably higher than the 20% rate for patients with 1-2 positive SLNs. Further analysis revealed that the number of positive SLNs was an independent predictor for nSLN metastasis, with patients having a 2.6-fold greater risk of nSLN metastases for each increase in the number of positive SLNs.

Hematological constants are thought to be a simple way to assess a patient’s systemic immunological and inflammatory condition (39). SII has been proposed as a factor with abilities to reflect distant metastasis, local recurrence and prognosis by outlining changes in platelets, neutrophils and lymphocytes in the circulatory system (40). In our study, elevated SII served as an independent predictor for nSLN metastasis following positive SLNs.

Furthermore, certain other factors, while not yielding favorable findings in the multivariate analysis, are nonetheless instructive to us.

Tumor size is one of the most important factors in determining the surgical approach and assessing patient prognosis. In the study by Seung Ki Min et al, increased tumor size predicted increased number of lymph node metastases (41). Also, tumor size served as a significant predictor for nSLN metastases in SLN-positive patients in the study by A. M. Moorman et al. (31) The present study showed that patients with larger lesion size were more likely to develop SLN metastasis. And the cut-off value was 3.2 cm on ROC analysis. However, in further logistic regression and analysis for nSLN metastasis, lesion size did not show positive results. The reason for this phenomenon, we speculate, in addition to the limitations of the single-center sample, may also be the bias caused by the single-tracer method and the subjective choice of surgeons.

The presence of a functional estrogen-signaling pathway and a better prognosis are assumed to be linked to high expression of ER and PR. Our findings appeared to imply a trend that patients with higher ER and PR expression got a higher risk of lymph node metastasis, even though it was not statistically significant in the multivariate logistic analysis. This is counterintuitive. Other trials, not coincidentally, came to similar results (38, 42, 43). The link between ER, PR, and lymph node metastases needs to be further investigated.

Age has been shown to be closely related to the incidence of breast cancer and the biological behavior of tumor cells (44). Breast cancers in younger patients tend to have a worse immunophenotype, such as a higher histological staging and a lower expression of hormone receptors (45). In our study, the mean age of all patients was 51.8. And in both comparisons, patients in the metastatic group were younger than those in the non-metastatic group, which was consistent with the existing theory.

BMI was included in this study for analysis because obesity was thought to be linked to a worse prognosis for breast cancer patients (46). In both comparisons, patients in the metastatic group had a higher BMI than those in the non-metastasis group, according to the findings.

In this study, we investigated SLN and nSLN metastasis together, looking longitudinally at factors related to lymph node metastasis in breast cancer. However, there are some limitations. First, the size of the intraoperatively removed SLNs was thought to be an important factor linked to nSLN metastasis, but due to lacking of data, we were unable to incorporate it in our analysis (47). Second, we are unable to investigate prognosis and surgical side-effects of patients due to a paucity of follow-up data. Finally, this is a single-center retrospective study with a limited sample size, more large-scale and multi-center studies are needed.

## Conclusion

For clinically node-negative breast cancer patients, “high expression of Ki-67” and “lymphatic invasion” imply a higher risk of SLN metastasis; for SLN-positive patients, “increased number of positive SLN” and “increased SII” imply a higher risk of nSLN metastasis. For such patients, appropriate axillary lymph node management is necessary.

## Data availability statement

The raw data supporting the conclusions of this article will be made available by the authors, without undue reservation.

## Ethics statement

This study was approved by Ethics Committee of China Medical University (Approval number: AF-SOP-07-1.1-01). All methods were performed in accordance with the relevant guidelines and regulations. All enrolled patients supported this study and signed an informed consent form. 

## Author contributions

XW conceived this study. FJ was the director for the fund. YL and YF collected medical records and drafted manuscript. ZJ, MC, and XY assisted in revising the manuscript. All authors read and approved the final manuscript.

## Funding

This study was supported by National Natural Science Foundation of China (No. 82073282), China Postdoctoral Science Foundation (No. 2020M681018) and Natural Science Foundation of Liaoning Province-Doctoral Research Program (No. 2021-BS-115).

## Conflict of interest

The authors declare that the research was conducted in the absence of any commercial or financial relationships that could be construed as a potential conflict of interest.

## Publisher’s note

All claims expressed in this article are solely those of the authors and do not necessarily represent those of their affiliated organizations, or those of the publisher, the editors and the reviewers. Any product that may be evaluated in this article, or claim that may be made by its manufacturer, is not guaranteed or endorsed by the publisher.

## Abbreviations

SLNB, sentinel lymph node biopsy; cALND, completion axillary lymph node dissection; SLN, sentinel lymph nodes; nSLN, non-sentinel lymph node; SII, systemic inflammation index; ASCO, American Society of Clinical Oncology; ER, estrogen receptors; PR, progesterone receptors; HER2, human epidermal growth factor receptor 2; PLR, platelet lymphocyte ratio; NLR, neutrophil lymphocyte ratio; AUC, area under the curve; ROC, receiver operating characteristic; DFS, disease-free survival; OS, overall survival; NAC, neoadjuvant chemotherapy; BCS, breast conserving surgery; NCCN, national comprehensive cancer network.
